# Dry Gangrene Following the Use of Carbolic Acid

**Published:** 1872-11

**Authors:** 


### Dry Gangrene Following the Use of Carbolic Acid.

M. Poncet, an *interne* of the Lyons Hospital, records in the *Bulletin de Theraputique* (July 30th), a case which he considers rightly to convey a caution against the rash use of carbolic acid by non-professional persons. A girl, aged 13, had her fore-finger injured by a splinter of wood, which penetrated under the nail. The end of the finger was dipped in a bottle of solution of carbolic acid, and a compress soaked in the acid was applied. The next day the part had a greyish color, and was insensible; and when M. Olliver first saw her, a week afterwards, mortification had extended as far as the upper third of the second phalanx, and a line of demarkation was being formed. The finger assumed a mummified appearance, and separated on the the thirty-fith day. This occurrence suggested to M. Olliver the possibility of amputating fingers and toes by means of the application of carbolic acid; and experiments were accordingly made by M. Viennois on rabbits and fowls. The results showed that the application of carbolic acid was liable to be followed by toxic symptoms; but that these might be prevented by the application of a ligature. M. Olliver endeavored to put the plan into execution in a case of disease of the great toe, by plunging it for some minutes in a concentrated solution of carbolic acid; the thickness of the epidermis, however, prevented mortification from being produced. M. Poncet records also the case of a man aged 23, who, having wounded the end of one of his fingers, applied for ten days charpie impregnated with carbolic acid, and afterwards poaltices. When he applied to M Olliver, two months after the accident, the terminal phalanx was in a state of dry gangrene, and was removed a few days afterwards.—*Medical Reporter*

